# Social Disparities of Pain and Pain Intensity Among Women Diagnosed With Early Stage Breast Cancer

**DOI:** 10.3389/fonc.2022.759272

**Published:** 2022-02-08

**Authors:** Hyo Young Choi, Ilana Graetz, Arash Shaban-Nejad, Lee Schwartzberg, Gregory Vidal, Robert Lowell Davis, Eun Kyong Shin

**Affiliations:** ^1^ Department of Preventive Medicine, University of Tennessee Health Science Center, Memphis, TN, United States; ^2^ Department of Medicine, University of Tennessee Health Science Center, Memphis, TN, United States; ^3^ Department of Health Policy & Management, Rollins School of Public Health, Emory University, Atlanta, GA, United States; ^4^ UTHSC-Oak Ridge National Lab (ORNL) Center for Biomedical Informatics, Department of Pediatrics, College of Medicine, University of Tennessee Health Science Center, Memphis, TN, United States; ^5^ West Cancer Center, Memphis, TN, United States; ^6^ Department of Hematology/Oncology, University of Tennessee Health Science Center, Memphis, TN, United States; ^7^ Department of Sociology, Korea University, Seoul, South Korea

**Keywords:** breast cancer, social determinants of health, cancer disparities, pain treatment, social epidemiology

## Abstract

**Background:**

Breast cancer is one of the most commonly diagnosed cancers among women in the United States and pain is the most common side effect of breast cancer and its treatment. Yet, the relationships between social determinants of pain and pain experience/intensity remain under-investigated. We examined the associations between social determinants of pain both at the individual level and the neighborhood level to understand how social conditions are associated with pain perception among early stage breast cancer patients.

**Methods:**

We conducted integrated statistical analysis of 1,191 women with early stage breast cancer treated at a large cancer center in Memphis, Tennessee. Combining electronic health records, patient-reported data and census data regarding residential address at the time of first diagnosis, we evaluated the relationships between social determinants and pain perception. Pain responses were self-reported by a patient as a numerical rating scale score at the patient’s initial diagnosis and follow-up clinical visits. We implemented two sets of statistical analyses of the zero-inflated Poisson model and estimated the associations between neighborhood poverty prevalence and breast cancer pain intensity. After adjustment for demographic characteristics, cancer stage, and chemotherapy, pain perception was significantly associated with poverty and blight level of the neighborhood.

**Results:**

Among women living in the highest-poverty areas, the odds of reporting pain were 2.48 times higher than those in the lowest-poverty area. Women living in the highest-blight area had 5.43 times higher odds of reporting pain than those in the lowest-blight area. Neighborhood-level social determinants were significantly associated with pain intensity among women diagnosed with early-stage breast cancer.

**Conclusions:**

Distressed neighborhood conditions are significantly associated with higher pain perception. Breast cancer patients living in socio-economically disadvantaged neighborhoods and in poor environmental conditions reported higher pain severity compared to patients from less distressed neighborhoods. Therefore, post-diagnosis pain treatment design needs to be tailored to the social determinants of the breast cancer patients.

## Introduction

Breast cancer is one of the most commonly diagnosed cancers among women in the United States. Pain is one of the common side effects of breast cancer and its treatment (1–3). Adequate pain management is critical to support treatment tolerance and quality of life (4–11). Patients’ quality of life is critical in improving cancer care (11). Pain among breast cancer patients is significantly associated with total life stress (12). As with the growing interest in the relationship between social conditions and health outcomes (13–17), in the present study, we investigated the social determinants of pain (18, 19) among breast cancer patients (20). It has been shown that social conditions critically influence health outcomes – chronic disease risks and mortality - at the individual level (21–24). The relationship between social conditions and perceived pain has not yet been rigorously investigated in a large number of breast cancer patients.

Abundant evidence has emphasized the importance of social conditions of breast cancer patients (6, 7, 11, 25–28). Living conditions of patients influence their accessibility to different breast cancer treatments (20, 29) and localized relative risk of breast cancer (30). The socioeconomic disparities among breast cancer patients (31, 32) signal an urgent need to address the concerns related to social determinants of pain. It has been reported that racial minorities and socially disadvantaged populations have higher prevalence of breast cancer (32) and low survival rates (31). To investigate these factors, we examined the social determinants of pain among breast cancer patients, expanding the current socioeconomic conditions both at the individual level and the neighborhood level. We scrutinized how multiple layers of socioeconomic features are associated with pain perception and aimed to answer the following questions. 1) How are social conditions of breast cancer patients at the individual level and at the neighborhood level associated with their pain perception? 2) Does distressed living condition influence breast cancer pain?

We merged the clinical data from electronic health records, patient-reported pain, and socio-economic information about the neighborhood of 1,191 women with breast cancer. By integrating multiple sources of data, we had an opportunity to explore and unveil the complexity of social embeddedness and perceived pain among breast cancer patients.

## Methods

### Data Sources

We examined pain reported by female patients diagnosed with early stage (I-III) hormone receptor-positive breast cancer at the West Cancer Center and Research Institute (WCCRI in Memphis, Tennessee. WCCRI is a comprehensive oncology center that collects patient-reported outcomes at each clinic visit using the Patient Care Monitor (PCM). The PCM is an electronic, tablet-based patient engagement platform used to collect information on patient-reported treatment side effects, physical and emotional symptoms, and functional status at the point of care. Patient responses are included in their electronic health record. Summary reports of patient PCM responses highlight significant changes as well as elevated symptom severity that may require further evaluation and treatment during the clinical visit (33). WCCRI has nine clinics and over 70 physicians providing a network of fully integrated cancer care that serves the tri-state area of west Tennessee, north Mississippi, and east Arkansas. WCCRI provides oncologic treatment for over 70% of all patients in the region and serves a diverse patient population.

### Population

We included female patients diagnosed with early stage (I-III) hormone receptor-positive breast cancer between 2007 and 2015 at the WCCRI. We merged the breast cancer patient data from the WCCRI and the 2010 census data including patients’ residential addresses at the time of their first diagnosis (N=3,835). According to the 2010 census, the overall population density in Tennessee was 162.9 residents per square mile and the Shelby County population density was 1,215.5 people per square mile (34). To maintain relative consistency of the setting, we limited the physical geography to Shelby County where the majority of the WCCRI patients reside. The neighborhood boundary can operate distinctively and an account of the impact of physical geography should be tailored accordingly. Therefore, we used 34 Zip codes within Shelby County, Tennessee, (N=2,584) to match the neighborhood-level data with the WCCRI data. The other inclusion criteria were availability of pain perception data and address information of the patients at the time of diagnosis. We also excluded one Zip code area that contained only two patients. Thus, we reduced the data set to patients with no missing data related to the main variables. Our final sample included 1,191 patients who reported their pain intensity at least once within 365 days since their initial diagnosis as well as patients from residential areas for which the data regarding neighborhood poverty prevalence were available (33 Zip code areas). This study has been reviewed and approved by the University of Tennessee Health Science Center Institutional Review Board, and waiver of patient consent was granted for the retrospective study.

### Variables and Measures

Descriptive statistics are shown in **Table 1**. We conducted group difference tests for each individual-level covariate and the significance levels of the differences were tested with a Wilcoxon test (35) for two-class variables and with a Kruskal-Wallis test (36, 37) for the variables with more than two classes (**Table 1**). Outcome variable is the self-reported pain and patients were asked to rate their pain-related experiences as numerical rating scale scores (from 0 to 10), with higher scores indicating severe perceived pain. This self-reported pain can be regarded as a comprehensive estimate of the patient’s complex pain perception associated with physical distress related to multiple symptoms. The patients were followed up during 6 years on average and reported their pain intensity at their initial diagnosis and subsequent clinical visits.

**Table 1:** Baseline characteristics related to pain intensity (nonparametric rank test).

	No. (%) (N=1,191)	Pain, mean [Q1, Q3]	*P*-value
**Response**			
** Pain**			
Mean (SD)	2.05 (2.46)		
Median [min, max]	1 [0, 10]		
**Neighborhood-level sociomarkers**			
** Poverty**			
Mean (SD)	21.56 (12.83)	1.02	<0.001
Median [min, max]	18.84 [2.47, 61.47]		
** Blight**			
Mean (SD)	13.85 (6.25)	1.03	<0.001
Median [min, max]	12.86 [3.24, 55.13]		
**Individual-level sociomarkers**			
Employment*			
Employed	463 (38.9%)	1.95 [0, 3]	0.016
Retired	66 (5.5%)	1.76 [0, 2.75]	
Unemployed	69 (5.8%)	2.93 [0, 5]	
Marital status*			
Married	413 (34.7%)	1.7 [0, 3]	0.004
Single	265 (22.3%)	2.41 [0, 4]	
Divorced	138 (11.6%)	2.3 [0, 4]	
Insurance type*			
Insurance	532 (44.7%)	1.9 [0, 3]	0.047
Medicaid	289 (24.3%)	2.29 [0, 4]	
**Biomarkers**			
** Cancer stage**			
1	667 (56%)	1.74 [0, 3]	<0.001
2	390 (32.7%)	2.45 [0, 4]	
3	134 (11.3%)	2.44 [1, 4]	
** Chemotherapy**			
Treated	624 (52.4%)	2.29 [0, 4]	<0.001
Untreated	567 (47.6%)	1.79 [0, 3]	
**Demographics**			
Race*			
White	634 (53.2%)	1.67 [0, 3]	<.001
Black	510 (42.8%)	2.55 [0, 4]	
** Age**			
Low	791 (66.4%)	2.16 [0, 4]	0.052
High	400 (33.6%)	1.83 [0, 3]	

SD, standard deviation; Min, minimum; max, maximum; Q1, 25^th^ percentile; Q3, 75^th^ percentile.

The baseline characteristics related to the included variables are summarized using mean with standard deviation and median with minimum and maximum values (min, max) for continuous variables and percentages for categorical variables. Along with the mean pain perception, the 25^th^ and 75^th^ percentiles [Q1, Q3] for each variable. There were missing values in some of the important variables such as employment, marital status, insurance type, neighborhood poverty and blight level. Missing values in employment, marital status, and insurance type were designated as “unknown”. However, patients with missing data regarding poverty and blight were excluded. For poverty and blight, we reported the estimates from Poisson regression for the association with pain intensity.

*indicates variables with NA’s. Employment (593 NA’s), Marital status (375 NA’s), Insurance type (370 NA’s), Race (47 NA’s).

To overcome the high noise and complexity in the outcome variable, we computed the average pain score for each patient during the observation period (from the initial diagnosis to 365 days after the initial diagnosis). Immediate pain (38) (within 3 months) (26, 39) and persistent pain (2) (during 3 years) have been important topics of discussion in many studies involving cancer patients, especially breast cancer patients (8). We focused on mean pain perception for a year after the initial diagnosis. The average number of pain reports is 5.8 (median = 4.0). Patients suffering from pain visit the clinic center more often compared to the patients with no pain shown by the significant association between positive pain perception and the number of pain reports (t-test, P < 0.001). Yet, the number of visits did not significantly differ by pain intensity within the patients who reported positive pain. 

Social determinants were measured both at the individual level and at the neighborhood level. Neighborhood-level social conditions have been suggested as key factors to study how social inequalities are engraved into health inequalities (16, 40–44). We measured the neighborhood social determinants using poverty level and blight prevalence from the US census data. Poverty [the percentage of individuals below the Federal Poverty Level (FPL) within a Zip code area (45)] is an indicator of socio-economic well-being of the neighborhood. Blight (the proportion of abandoned and vacant housing units within the neighborhood) is an indicator of the quality of built environment, which is considered a stressor that negatively affects the quality of life (46, 47) and various health outcomes (24, 46, 48). Due to their high correlation, blight and poverty were analyzed separately in different models. Individual-level social determinants were measured using employment status, marital status, and insurance type (Commercial Insurance vs Medicaid) provided by the WCCRI data. For the analysis of treatment types, we included chemotherapy, which has been suggested as a possible source of chronic pain among breast cancer survivors (1). Demographic variables [age (49, 50) and race (9)] and socio-economic class (51) are known to be associated with pain-related experiences and cancer stages, which were incorporated into the analysis.

### Statistical Analyses

We estimated the associations between social determinants and pain with the final models adjusted for multiple individual-level covariates including biomarkers (cancer stage, chemotherapy) and demographic attributes (age and race). To examine the association between social determinants and pain, we conducted group difference tests to examine the significance of differences among different categories of independent variables. We also investigated the impact of these variables on pain intensity in an adjusted as well as unadjusted regression framework. In the unadjusted models, we analyzed pairwise regression of pain intensity against each of the covariates, revealing partial relationships. The adjusted model included all available regressors selected by model selection.

A large proportion of patients reported no perceived pain, resulting in a considerable number of zero counts in the response variable. Altogether, 452 patients (39.5%) reported pain. Considering a standard Poisson model, there were significantly more number of zero counts than expected (*P*<0.001). To deal with the excess zero counts, we applied the zero-inflated Poisson (ZIP) model, which fit the zero count component using a logit model and the count component using a Poisson model (52–55). The ZIP model introduces additional probability weight for zero counts to generalized linear models (GLMs). Thus, data are modeled using a two-component mixture (Poisson model for the count component and a point mass at zero), which resulted in a significant model improvement by fitting the ZIP model when compared with a standard Poisson GLM [Vuong test (56, 57) (*P*<0.001)]. We additionally tested and confirmed that the assumptions of the ZIP model were valid based on comprehensive model diagnostic tests including linearity, link function, response variable distribution, outliers, and multicollinearity. The results from the ZIP model were reported separately for each component. The outputs from the Poisson count data component were reported using the estimated incidence rates (exponential transformation of the estimates) and the outputs from the zero-inflation component were reported using the estimated odds ratios.

While selecting variables for the adjusted model, we started from a fully specified model including all interaction terms among the social determinant variables and other variables, and performed a backward variable selection procedure. We tested only the main covariates, namely poverty, blight, and individual-level social context variables for model selection with the other covariates fixed in the model. All statistical analyses were performed using R (version 4.0.0, the R Foundation, Vienna, Austria) and the pscl package was used to fit the zero-inflated model. All of the methods were performed in accordance with the Declaration of Helsinki and the relevant guidelines.

## Results

Applying the inclusion criteria described above, a total of 1,191 women with breast cancer aged 22 to 95 years participated (791 patients with low age (age < 65) [66.4%]; 634 Whites [53.2%], 510 African Americans [42.8%]; 667 stage I [56%], 390 stage II [32.7%], 134 stage III [11.3%]). The averages for neighborhood-level social determinants of pain from 33 zip code areas were 21.56 for poverty and 13.85 for blight levels. For individual-level social determinants of pain, 38.9% of participants were employed (Retired [5.5%], Unemployed [5.8%]), 34.7% were married (Single [22.3%], Divorced [11.6%]), and 44.7% had commercial insurance (Medicaid [24.3%]). Mean values of pain intensity, by individual and neighborhood-level characteristics, are presented in **Table 1** with the association between pain intensity and each covariate. Levels of pain intensity significantly differed according by sociomarker at the neighborhood-level and at the individual-level and also according to other variables including cancer stage, chemotherapy, and race. Patients residing in areas with higher poverty and higher blight reported higher pain severity. In addition, among the individual-level social context variables, married patients (34.7%) reported higher pain than single patients (22.3%) (*P*<0.001) and unemployed patients (5.8%) perceived higher pain intensity than employed patients (38.9%) and retired patients (5.5%) (*P*=0.016). Medicaid beneficiaries (24.3%) reported significantly higher pain intensity than patients with other types of insurance (44.7%) (*P*=0.047). Higher pain perception was associated with higher cancer stage at diagnosis (*P*<0.001) and patients treated with chemotherapy reported higher pain perception (*P*<0.001). We also observed higher pain perception among African American patients compare to white patients (*P*<0.001).

### Pain Occurrence

**Table 2** has summarized the association between the absence of pain and the covariates after exponentiating the estimates, which can be interpreted as odds ratios (ORs) relative to the reference classes. Cancer stage showed the most substantial association with the probability of pain occurrence (*P*<0.001). With progression in the cancer stage, patients were more likely to suffer from pain. Moreover, patients who did not undergo chemotherapy had a lower probability of reporting any pain compared to patients treated with chemotherapy (*P*<0.001).

**Table 2:** Inflation model: no pain vs. pain.

	Unadjusted (N=1,191)			Adjusted (N=1,191)			Adjusted (N=1,191)		
	Estimate	95% CI	P-value	Estimate	95% CI	P-value	Estimate	95% CI	P-value
(Intercept)				1.139	0.767-1.692	0.52	1.004	0.651-1.548	0.987
**Poverty**	1.018	1.008-1.029	<.001	1.015	1.003-1.028	0.011			
**Blight**	1.037	1.016-1.058	<.001				1.033	1.008-1.057	0.008
**Occupation**									
Employed (reference)									
Retired	1.12	0.614-2.041	0.711	0.996	0.503-1.972	0.991	0.99	0.5-1.961	0.978
Unemployed	1.353	0.772-2.37	0.29	0.862	0.465-1.595	0.636	0.86	0.464-1.592	0.631
Unknown	0.967	0.742-1.263	0.808	0.757	0.53-1.082	0.127	0.755	0.528-1.079	0.123
**Marital status**									
Married (reference)									
Single	1.188	0.852-1.656	0.311	0.856	0.587-1.248	0.419	0.86	0.59-1.252	0.431
Divorced	1.6	1.032-2.488	0.036	1.431	0.892-2.294	0.137	1.429	0.89-2.288	0.14
Unknown	1.309	0.961-1.783	0.088	2.222	0.951-5.181	0.065	2.193	0.94-5.128	0.069
**Insurance type**									
Commercial Insurance (reference)									
Medicaid	1.397	1.017-1.916	0.039	1.779	1.164-2.717	0.008	1.776	1.161-2.71	0.008
Unknown	1.202	0.899-1.605	0.214	0.776	0.337-1.786	0.551	0.784	0.341-1.802	0.566
**Cancer stage**									
1 (reference)									
2	1.866	1.412-2.463	<.001	1.605	1.176-2.183	0.003	1.616	1.185-2.198	0.002
3	3.077	1.876-5.051	<.001	2.747	1.511-4.975	0.001	2.786	1.529-5.051	0.001
**Chemo**									
Treated (reference)									
Untreated	0.512	0.398-0.659	<.001	0.619	0.466-0.823	0.001	0.621	0.467-0.826	0.001
**Race**									
White (reference)									
African American	1.497	1.155-1.942	0.002	1.031	0.75-1.416	0.853	1.055	0.775-1.437	0.732
Unknown	0.704	0.381-1.3	0.262	0.707	0.37-1.35	0.293	0.71	0.372-1.353	0.298
**Age**									
Low (reference)									
High	0.917	0.704-1.193	0.519	0.786	0.558-1.105	0.166	0.785	0.558-1.105	0.165

CI, confidence intervals.

We found significant association between no pain and three social determinant- poverty, blight, and insurance types. In the unadjusted model, the ORs of the estimated probabilities of pain occurrence and no pain were 1.015 for every 1-unit increase in poverty (95% confidence interval [CI], 1.003–1.028) and 1.033 for every 1-unit increase in the blight level (95% CI, 1.008–1.057). These results suggest that the odds of perceiving pain in the highest-poverty area and in the highest-blight area were expected to increase by about 140% and 600%, respectively, when compared with the lowest-poverty area and the lowest-blight area. The estimated odds of the presence of pain in the Medicaid group were 1.78 times higher (Unadjusted OR: 1.397; 95% CI, 1.017–1.916 and Adjusted OR, 1.779; 95% CI, 1.164–2.717; respectively). The estimated probabilities of zero pain in the commercial insurance group and in the Medicaid group at fixed levels of other covariates (mean values for poverty and blight, reference values for the others) were 0.39 (95% CI, 0.25–0.55) and 0.26 (95% CI, 0.11–0.51), respectively, demonstrating a considerable inequality.

### Pain Intensity

**Table 3** demonstrates the association between pain intensity and the covariates through count components of the ZIP regression analysis. Areas with higher poverty and blight were significantly associated with greater pain intensity in both unadjusted and adjusted models. The incidence rate (IR) of neighborhood poverty in the unadjusted model was 1.01 (95% CI, 1.007–1.013), which means 1% of increase in the neighborhood poverty, the expected pain intensity increased by 1%. Pain perception was expected to increase by 81.6% in the highest-poverty area compared to the lowest-poverty area considering the range of the observed poverty variable (range: 2–61%) in our cohort (in the unadjusted model IR, 1.016; 95% CI, 1.01–1.023). Pain intensity in the highest-blight area was 2.58 times the pain intensity in the lowest-blight area. Even after adjustment for other covariates, the results were consistent with IR of 1.006 (95% CI, 1.002–1.009) for poverty and 1.007 (95% CI, 1–1.015) for blight. These findings showed that 43.2% and 43.7% increases in pain intensity were expected in the highest-poverty area and in the highest-blight area, respectively, when compared with the areas with the lowest poverty and blight.

**Table 3:** Count model: pain intensity.

	Unadjusted (N=1,191)			Adjusted (N=1,191)			Adjusted (N=1,191)		
	Estimate	95% CI	P-value	Estimate	95% CI	P-value	Estimate	95% CI	P-value
(Intercept)		NA-NA		2.203	1.892–2.565	<.001	2.204	1.876–2.59	<.001
**Poverty**	1.01	1.007–1.013	<0.001	1.006	1.002–1.009	0.004			
**Blight**	1.016	1.01–1.023	<0.001				1.007	1–1.015	0.046
**Occupation**									
Employed (reference)									
Retired	0.863	0.696–1.069	0.178	0.868	0.688–1.089	0.221	0.868	0.692–1.089	0.222
Unemployed	1.35	1.152–1.583	<0.001	1.335	1.128–1.58	0.001	1.345	1.137–1.592	0.001
Unknown	1.065	0.971–1.168	0.182	1.106	0.984–1.244	0.092	1.111	0.988–1.25	0.078
**Marital status**									
Married (reference)									
Single	1.32	1.177–1.48	<0.001	1.204	1.065–1.36	0.003	1.22	1.08–1.378	0.001
Divorced	1.137	0.986–1.311	0.078	1.095	0.948–1.266	0.216	1.092	0.945–1.261	0.233
Unknown	1.105	0.989–1.234	0.077	1.004	0.777–1.298	0.976	0.993	0.768–1.272	0.917
**Insurance type**									
Commercial Insurance (reference)									
Medicaid	1.063	0.957–1.18	0.253	1.073	0.947–1.215	0.269	1.07	0.944–1.212	0.288
Unknown	1.018	0.92–1.125	0.731	1.096	0.855–1.406	0.47	1.108	0.864–1.421	0.419
**Cancer stage**									
1 (reference)									
2	1.118	1.021–1.225	0.017	1.05	0.951–1.16	0.332	1.052	0.953–1.162	0.315
3	0.978	0.857–1.117	0.746	0.861	0.746–0.994	0.042	0.862	0.746–0.996	0.043
**Chemotherapy**									
Treated (reference)									
Untreated	1.01	0.926–1.101	0.824	1.066	0.97–1.172	0.186	1.065	0.969–1.171	0.189
**Race**									
White (reference)									
African American	1.319	1.208–1.439	<0.001	1.202	1.084–1.333	0.001	1.235	1.117–1.367	<0.001
Unknown	1.271	1.006–1.606	0.045	1.164	0.916–1.479	0.214	1.176	0.926–1.494	0.184
**Age**									
Low (reference)									
High	0.873	0.795–0.959	0.005	0.873	0.784–0.973	0.014	0.877	0.787–0.977	0.018

CI, confidence intervals.

Among the individual social context variables, employment and marital status were significantly associated with lower pain intensity. In the unadjusted models, unemployed patients showed 35.5% greater pain perception than the employed group [IR, 1.35; 95% CI, 1.152–1.583, in the adjusted model IR 1.335 (95% CI:1.128–1.58)]. Single patients showed 32% greater pain than the married group (IR, 1.32; 95% CI, 1.177–1.48). The adjusted model exhibited similar results with a slightly lower IR of 1.204 (95% CI, 1.065–1.36).

## Discussion

We observed that markers of lower socio-economic status were associated with higher self-reported pain perception among breast cancer patients in Memphis, Tennessee. After adjustment for demographic characteristics, cancer stage, chemotherapy, and individual-level social determinants, patients living in areas with higher levels of poverty or blight were more likely to report any pain and higher pain severity. We also observed a strong association between individual-level social determinants and pain experience. Marital status and employment status showed significant associations with pain severity. Insurance type showed significant association with pain perception.

The importance of social embeddedness with respect to mortality and quality of life in breast cancer is widely known (6, 7, 11, 25–28). Our results are consistent with the results of previous studies investigating the associations between social embeddedness of breast cancer patients and cancer prevalence and survival rates. Some studies have shown that individual-level social condition was associated with breast cancer mortality. Socially isolated patients had increased risk of breast cancer mortality compared to socially integrated patients (26–28). Other studies have demonstrated the effects of neighborhood-level socio-economic markers (race, ethnicity, and living area) (29, 32) on the disparities in breast cancer stage and mortality. Our data expanded the social determinants both at the individual level and at the neighborhood level by inclusion of a large and racially well-balanced cohort. To the best of our knowledge, this is the first study to report an association between breast cancer pain perception and social determinants of pain at the neighborhood level and at the individual level. We also found that race, cancer stage, and chemotherapy were important factors in breast cancer pain perception, which is consistent with previous results. However, our distinctive contribution is in uncovering the associations between the pain perception and both individual and neighborhood-level social determinants even after adjusting for the other well-known factors.

Neighborhoods are important in influencing individual health outcomes (16, 17). Consistent with previous studies, we observed a variance in patients’ pain perception during the first year after diagnosis and 60% of the patients did not report any pain. While biomarker-related indicators, cancer stage, and chemotherapy were not significantly associated with pain perception, social determinants of pain at the individual level and neighborhood level showed significant association with pain perception. The burden of breast cancer is disproportionately distributed across different social strata and social conditions are significantly associated with the most common side effects of breast cancer. Women living in poorer neighborhoods may be under-treated for pain. Therefore, the post-diagnosis treatment design needs to be tailored to the social determinants of the breast cancer patients. Future studies should include cost of pain medication or increasing likelihood of denial in Medicaid coverage versus private to further identify the deeper mechanisms of the pain disparities.

The study has some limitations. All the participants were from a single location (Memphis, Tennessee). The generalizability of the findings might depend on the local setting. The high prevalence of poverty and the unique demographics (52.1% African American and 40.6% Caucasian) in Shelby County, Tennessee should be considered while applying our findings to other states. Our residential address linking procedure had its own limitations. We did not have information regarding residence before the diagnosis. Thus, we could not determine if the patients lived in the neighborhood long enough to be influenced by its local condition. We merged the data sets at the time of the first diagnosis. Due to the lack of data availability, we could not control the prior residential history. Additionally, we do not have medication data, which would have been helpful to further understanding of the mechanism of pain disparities. Future studies should include pharmaceutical data, which can offer a better care plan those paints living in disadvantaged neighborhoods.

Despite the limitations, the findings strongly suggest that breast cancer patients living in socio-economically disadvantaged neighborhoods exhibited higher intensity of pain when compared with patients living in less distressed neighborhoods. We observed that the disparities in pain experience depended on the neighborhood-level social conditions. During post-diagnosis pain management in breast cancer patients, the patient care plan should be consider not only to the pathological factors but also the social conditions of patients for better-tailored pain and disease management.

## Data Availability Statement

The data analyzed in this study is subject to the following licenses/restrictions: Although electronic health record data will not be available due to patient privacy, the R codes that support the findings of this study are available on Github. (https://github.com/hyochoi/BreastCancerPainStudy). Requests to access these datasets should be directed to Ilana Graetz, ilana.graetz@emory.edu.

## Ethics Statement

This study was approved by the University of Tennessee Health Science Center institutional review board and participant written informed waiver of patient consent was granted for the retrospective study (IRB 17-05479-XP IA).

## Author Contributions

ES conceptualized, designed the study. HC and ES analyzed the data, wrote the original draft. LS, GV, and IG provided the data. IG curated and cleaned the data. All authors revised the manuscript and approved this version.

## Funding

This research was supported in part by NCI Health Grant (R21CA208161) and Korea University Future Research Grant (K2007651). Funders did not have any role in the study design; collection, analysis, or interpretation of data; the writing of the manuscript; or the decision to submit the manuscript for publication.

## Conflict of Interest

The authors declare that the research was conducted in the absence of any commercial or financial relationships that could be construed as a potential conflict of interest.

## Publisher’s Note

All claims expressed in this article are solely those of the authors and do not necessarily represent those of their affiliated organizations, or those of the publisher, the editors and the reviewers. Any product that may be evaluated in this article, or claim that may be made by its manufacturer, is not guaranteed or endorsed by the publisher.
